# Partial mixture model for tight clustering of gene expression time-course

**DOI:** 10.1186/1471-2105-9-287

**Published:** 2008-06-18

**Authors:** Yinyin Yuan, Chang-Tsun Li, Roland Wilson

**Affiliations:** 1 Department of Computer Science, University of Warwick, Coventry, UK

## Abstract

**Background:**

Tight clustering arose recently from a desire to obtain tighter and potentially more informative clusters in gene expression studies. Scattered genes with relatively loose correlations should be excluded from the clusters. However, in the literature there is little work dedicated to this area of research. On the other hand, there has been extensive use of maximum likelihood techniques for model parameter estimation. By contrast, the minimum distance estimator has been largely ignored.

**Results:**

In this paper we show the inherent robustness of the minimum distance estimator that makes it a powerful tool for parameter estimation in model-based time-course clustering. To apply minimum distance estimation, a partial mixture model that can naturally incorporate replicate information and allow scattered genes is formulated. We provide experimental results of simulated data fitting, where the minimum distance estimator demonstrates superior performance to the maximum likelihood estimator. Both biological and statistical validations are conducted on a simulated dataset and two real gene expression datasets. Our proposed partial regression clustering algorithm scores top in Gene Ontology driven evaluation, in comparison with four other popular clustering algorithms.

**Conclusion:**

For the first time partial mixture model is successfully extended to time-course data analysis. The robustness of our partial regression clustering algorithm proves the suitability of the combination of both partial mixture model and minimum distance estimator in this field. We show that tight clustering not only is capable to generate more profound understanding of the dataset under study well in accordance to established biological knowledge, but also presents interesting new hypotheses during interpretation of clustering results. In particular, we provide biological evidences that scattered genes can be relevant and are interesting subjects for study, in contrast to prevailing opinion.

## Background

Based on the assumption that co-expression indicates co-regulation, gene expression data clustering aims to reveal gene groups of similar functions in the biological pathways. This biological rationale is readily supported by both empirical observations and systematic analysis [1]. In particular, consider gene expression time-course experiments, where the data are made up of tens of thousands of genes, each with measurements taken at either uniformly or unevenly distributed time points often with several replicates. Clustering algorithms provide a good initial investigation into such large-scale datasets, which ultimately leads to biological inference. An excellent review of current techniques and all subsequent analysis can be found in [2].

Various model-based methods have been proposed to accommodate the needs for data mining in such massive datasets. Among them are mixed effects models [3,4] and auto regressive models [5]. The basic approach of these model-based methods is to fit a finite mixture model to the observed data, assuming that there is an underlying true model/density, and then systemically find the optimal parameters so that the fitted model/density is as close to the true model/density as possible. It is observed that model-based approaches generally achieve superior performance to many others [6-9]. However, current methods can be problematic, as they often fail to show how clustering can assist in mining gene expression data.

The maximum likelihood estimator (MLE) is one of the most extensively used statistical estimation techniques in the literature. For a variety of models, likelihood functions [4,6,10], especially maximum likelihood, have been used for making inferences about parameters of the underlying probability distribution for a given dataset. The solution often involves a nonlinear optimization such as quasi-Newton methods or, more commonly, expectation-maximization (EM) methods [4,11]. The problem with the former method is that the quantities are estimated only when they satisfy some constraints, while with the latter method all parameters have to be explicitly specified, so the number of clusters *K *has to be known a priori, which is not practical in microarray data analysis. There are many unique features of MLE, including its efficiency. However the practical deficiencies of MLE, besides those with its optimization, are the lack of robustness against outliers and its sensitivity to the correctness of model specification. We discuss in this paper the performance of an appealing alternative, the minimum distance estimator (MDE) [12], which is less explored in this field. Inspired by the work of [13], we propose to incorporate MDE in our algorithm for gene expression time-course analysis. MDE provides robust estimation against noise and outliers, which is of particular importance in gene expression data analysis, where data are often noisy and there are few replicates.

Tight clustering has been proposed as a response to the needs for obtaining smaller clusters in genomic signal processing. It was motivated by the fact that the most informative clusters are very often the tight clusters, usually of size 20–60 genes [14]. Tight clustering refers to methods that can be built upon an existing partition to obtain core patterns that are more interpretable. The initial partition can be obtained empirically or by using generic algorithms such as K-means. As a result, more information can possibly be revealed. For example, if genes in the same functional category are allocated into different tight clusters, one may pursue the underlying explanation by looking into these clusters. One possible result of such investigation, for example, is new function discovery.

In this sense, to obtain tight clusters, some genes should be classified as scattered genes, if forcing them into clusters will only disturb biologically relevant patterns. Indeed, the issue of scattered genes has received more attention recently [2,14]. However, in contrast to the prevailing concept that scattered genes should be treated as outliers and discarded from further study, we prove that some scattered genes can be of biological significance. Current methods for gene expression time-course data rarely deal with scattered genes. To the best of our knowledge, [14] is the first to address this issue, but it results in heavy computation due to the nature of random resampling. It was proposed in [11] that outliers can be modelled by adding a Poisson process component in the mixture model. However, this method has not been verified in this field, and it relies on correct model specification.

There has been a lot of research focusing on modelling time-course data by splines and autoregressive models, usually followed by EM [3,4,6,15,16]. In [15], the cubic B-spline, which is a linear combination of B-spline basis functions, is used for fitting gene expression time-course data. To avoid over-fitting, it is suggested not to fit a curve to every individual gene, but to constrain the spline coefficients of co-expressed genes to have the same covariance matrix. Alternatively, we propose in this work a novel approach to fit our spline model.

SplineCluster [6] is an efficient hierarchical clustering program based on a regression model with a marginal likelihood criterion. Starting from singleton clusters, the idea is to merge clusters based on marginal likelihood in each iteration. It is efficient and straightforward to visualize. The problem is that it overlooks microarray replication information by using only the mean of all replicates, which leads to loss of information. As microarry experiments are increasingly performed with replicates, the additional information provided by replicated measurements is a valuable source of variability in terms of effective clustering [17].

The outline of this paper is as follows. In the second section, we describe the MDE framework and demonstrate how its excellent properties inspire a partial regression model for fitting gene expression time-course data. Simulated datasets are designed for fitting by both partial MDE and MLE, to reveal their inherent differences. Built upon the advantages of MDE and partial modelling, a robust partial regression clustering algorithm is proposed for tight clustering which naturally incorporates replication information and allows a set of scattered genes to be left out. The experimental section is made up of two parts. First, our proposed partial regression clustering algorithm is applied to a simulated dataset to demonstrate its effectiveness. Secondly, it is compared with some recent work by applying the methods to two well studied real datasets. The superior performance of our algorithm is found through a carefully organized clustering validation, based on both biological knowledge and statistical indices. In particular, a Gene-Ontology (GO) [18] driven validation measure is proposed, specifically designed for gene expression clustering. Subsequent analysis of the clustering outcome reveals new knowledge generated by incorporating different biological resources. This study not only explores the differences between the two estimators and the application of partial modelling, but also provide an excellent example of gene expression data mining through the combination of machine learning and biological knowledge. Owning to space restrictions, some discussions, results and elaborations have been relegated [see Additional file 1, Section 1].

## Results and Discussion

### Minimum Distance Estimation and Partial Modelling

#### Minimum Distance Estimator (MDE)

Given a density function *f*(·), its corresponding parameters *θ *and *n *samples *x*_*i*_, *i *= 1, 2, ..., *n*, we aim to find the optimal parameters $\hat{\theta }$ to approximate the true parameters *θ*_0 _by minimizing the integrated squared difference

(1) *d*(*f*(*θ*), *f*(*θ*_0_)) = ∫ [*f*(*x*|*θ*) - *f*(*x*|*θ*_0_)]^2 ^*dx*

which gives

(2) *d*(*f*(*θ*), *f*(*θ*_0_)) = ∫ *f*(*x*|*θ*)^2 ^*dx *- 2 ∫ *f*(*x*|*θ*) *f*(*x*|*θ*) *f*(*x*|*θ*_0_)*dx *+ ∫ *f*(*x*|*θ*_0_)^2 ^*dx*

The last integral ∫ *f*(*x*|*θ*_0_)^2 ^*dx *is a constant with respect to *θ*, thus can be ignored. The second integral can be obtained through kernel density estimation [19]. Therefore, the MDE criterion simplifies to

(3) $$\hat{\theta }=\arg\underset{\theta }{\min}[\int f{\left(x|\theta \right)}^{2}dx-\frac{2}{n}\sum_{i=1}^{n}f(x_{i}|\theta )]$$

There are many interesting features of MDE. First of all, it comes with the same robustness as all other minimum distance techniques [20-23]. Secondly, MDE approximates data by making the residuals as close to normal in distribution as possible [20-22]. These features will be further explained and illustrated in the experiments. We will also illustrate derivation of the MDE criterion for parameter estimation for our partial regression algorithm.

#### Gaussian Mixture Model with MDE

In principle, the finite mixture model methodology assumes that the probability density function, *f*(*x*|*θ*), can be modelled as the sum of weighted component densities. The weights are often constrained to have a sum of 1. It is revealed later that this constraint is not necessary. More flexible models can be obtained by relieving the system from this constraint. A weighted Gaussian mixture model has the form:

(4) $$\begin{array}{cc} f(x|\theta )=\sum_{k=1}^{K}w_{k}\varphi (x|\mu_{k},\sigma_{k}^{2}), & w_{1}+w_{2}+\ldots w_{K}=1 \end{array}$$

where *φ *is the Gaussian density function, *μ*, *σ *are mean and standard deviation, *K *is the number of components, and *w*_*k*_, *k *= 1, 2, ..., *K *are the weight parameters. However, by relieving the constraint of $\sum_{k=1}^{K}w_{k}=1$ the system can be extended for overlapping clustering inference [13] since the sum of the amount of data being modelled in all clusters can exceed the total amount of data. Later, we will further prove that the amount of modelled data can also be less than the total amount of data. In all cases, *w*_*k *_indicates the proportion of data points that are allocated in the *k*th component. Let *g*_*K *_(*x*|*θ*) be the part in Eq.(3) to be minimized for a *K*-component mixture model, we have

(5) $$g_{K}(x|\theta )=\int f{\left(x|\theta \right)}^{2}dx-\frac{2}{n}\sum_{i=1}^{n}f(x_{i}|\theta )$$

On the other hand,

(6) ∫φ(x|μ,σ2)2dx=∫[12πσexp⁡(−(x−μ)22σ2)]2dx=12σπ∫12πσ2exp⁡(−(x−μ)22(σ2)2)dx=12σπ

And from Section 2.6 of [24]

(7) ∫φ1(x|μ1,σ12)φ2(x|μ2,σ22)dx=φ(μ1−μ2|0,σ12+σ22)∫φ(x|σ12μ2+σ22μ1σ12+σ22,σ12σ22σ12+σ22)dx=φ(μ1−μ2|0,σ12+σ22)

By combining Eq.(4), (6) and (7), we have

(8) ∫f(x|θ)2dx=∫(∑k=1Kwk2φk2+∑k=1K∑l=1Kwkwlφkφl)dx=∑k=1Kwk22πσk+∫∑k=1K∑l=1Kwkwlφkφldx=∑k=1Kwk22πσk+∑k=1K∑l=1Kwkwlφ(μk−μl|0,σk2+σl2)

Thus from Eq.(5) and (8) the distance for the *K*-component Gaussian mixture model can be expressed as:

(9) $$g_{K}(x|\theta )=\sum_{k=1}^{K}\frac{w_{k}^{2}}{2\sqrt{\pi }\sigma_{k}}+\sum_{k=1}^{K}\sum_{l=1}^{K}w_{k}w_{l}\varphi (\mu_{k}-\mu_{l}|0,\sigma_{k}^{2},\sigma_{l}^{2})-\frac{2}{n}\sum_{i=1}^{n}\sum_{k=1}^{K}w_{k}\varphi (x_{i}|\mu_{k},\sigma_{k}^{2})$$

*g*_*K *_(*x*|*θ*) is a closed-form expression, whose minimization can be performed by a standard nonlinear optimization method.

For example, a one-component model has the following MDE criterion:

(10) $$\begin{array}{lll} \hat{\theta } & = & \arg\underset{\theta }{\min}[g_{1}(x|\theta )]\\ & = & \arg\underset{\theta }{\min}[\frac{w^{2}}{2\sqrt{\pi }\sigma }-\frac{2w}{n}\sum_{i=1}^{n}\varphi (x_{i}|\mu ,\sigma^{2})] \end{array}$$

To further relieve the system from constraints by the weight parameters, while keeping its weighted-component structure, in the next section the idea of partial modelling is presented. It originated from the fact that incomplete densities are allowed [25], so the model will be fitted to the most relevant data.

### Partial Mixture Model with MDE (PMDE)

The weight parameters are of particular importance in a partial mixture model. They allow the model to estimate the component/components, while their value indicates the proportions of fitted data, so the rest of the data can be treated as scattered genes. This approach is first described in [13] for outlier detection. It was suggested to accommodate scattered genes by forcing a large scaling parameter in one of the components in the mixture [2]. However, partial modelling provides a better alternative.

Although it is suggested in [13] that the unconstrained mixture model can be applied for clustering, through our experiments it is clear that if the data overlap to a certain degree, all components will converge to the biggest component as a result of model freedom. Moreover, it is not practical to formulate the criterion in the form of Eq.(9) when it comes to implementation. Instead, we solve the problem by taking advantage of the one-component model to formulate our clustering algorithm.

#### Partial Regression Model

To analyse such high dimensional data as gene expression time-course measurements, a regression model with a cubic B-spline basis is set up in order to account for the inherent time dependence. The linear regression model is capable of handling either uniformly or unevenly distributed time points, while the nonlinear spline basis helps accommodate the underlying stochastic process in the data. The advantage of using cubic B-spline lies in that the degree of the polynomials is independent of the number of points and that curve shape is controlled locally. Let *Y *be the variables of interest, consisting of gene expression data replicate matrices modelled as

(11) *Y *= *α *+ *X*(*t*)*β *+ *ε*

*X*(*t*) is the design matrix consisting of a linear combination of cubic spline basis functions. The error term *ε *represents the residuals taken as a weighted distribution *w*·*N*(0, $\sigma_{\varepsilon }^{2}$). *α*, *β *= *β*_1_, *β*_2_, ..., *β*_*m*_, *m *depending on the choice of *X*(*t*), are the regression parameters. As stated before, the useful feature of MDE is that it fits data in such a way that the residuals are close to a normal distribution. Therefore our model is

(12) *ε *= *Y *- *α *- *X*(*t*)*β*

Therefore, given Eq.(4) and (6), the one-component PMDE fit for this model has the form of

(13) $$\begin{array}{lll} \hat{\theta } & = & \arg\underset{\theta }{\min}[\int (w\varphi (\varepsilon |0,\sigma_{\varepsilon }){)}^{2}d\varepsilon -\frac{2}{n}\sum_{i=1}^{n}w\varphi (\varepsilon_{i}|0,\sigma_{\varepsilon }^{2})]\\ & = & \arg\underset{\theta }{\min}[\frac{1}{2\sqrt{\pi }}w^{2}\sigma_{\varepsilon }^{-1}-\frac{2w}{n}\sum_{i=1}^{n}\varphi (\varepsilon_{i}|0,\sigma_{\varepsilon }^{2})] \end{array}$$

where *θ *= {*w*, *α*, *β*_1_, ...*β*_*m*_, *σ*_*ε*_} and *φ *is the density of a normal random variable. Altogether there are *m *+ 3 parameters to be estimated.

#### Simulated Datasets for PMDE fitting

The main feature of our model is that it is able to identify the key component, if any, and a set of outliers, in order to find the core structure. Therefore, a feasible parameter estimator is of paramount importance. We empirically validate our points about the nature of partial modelling and MDE through fitting four simple simulated datasets. The performance of both PMDE and MLE with a one-component spline regression model (*K *= 1) is compared in terms of data fitting accuracy and robustness. Surprisingly, superior performance was achieved for the PMDE fits even on such simple datasets. All datasets are generated by sine functions, modelling cyclic behavior of genes, which are widely employed in the literature [3,26]. Gaussian noise is added to all data. The number of knots for both spine models is chosen to be 15, to allow for flexibility in curves while avoiding overfitting.

**Figure 1:**
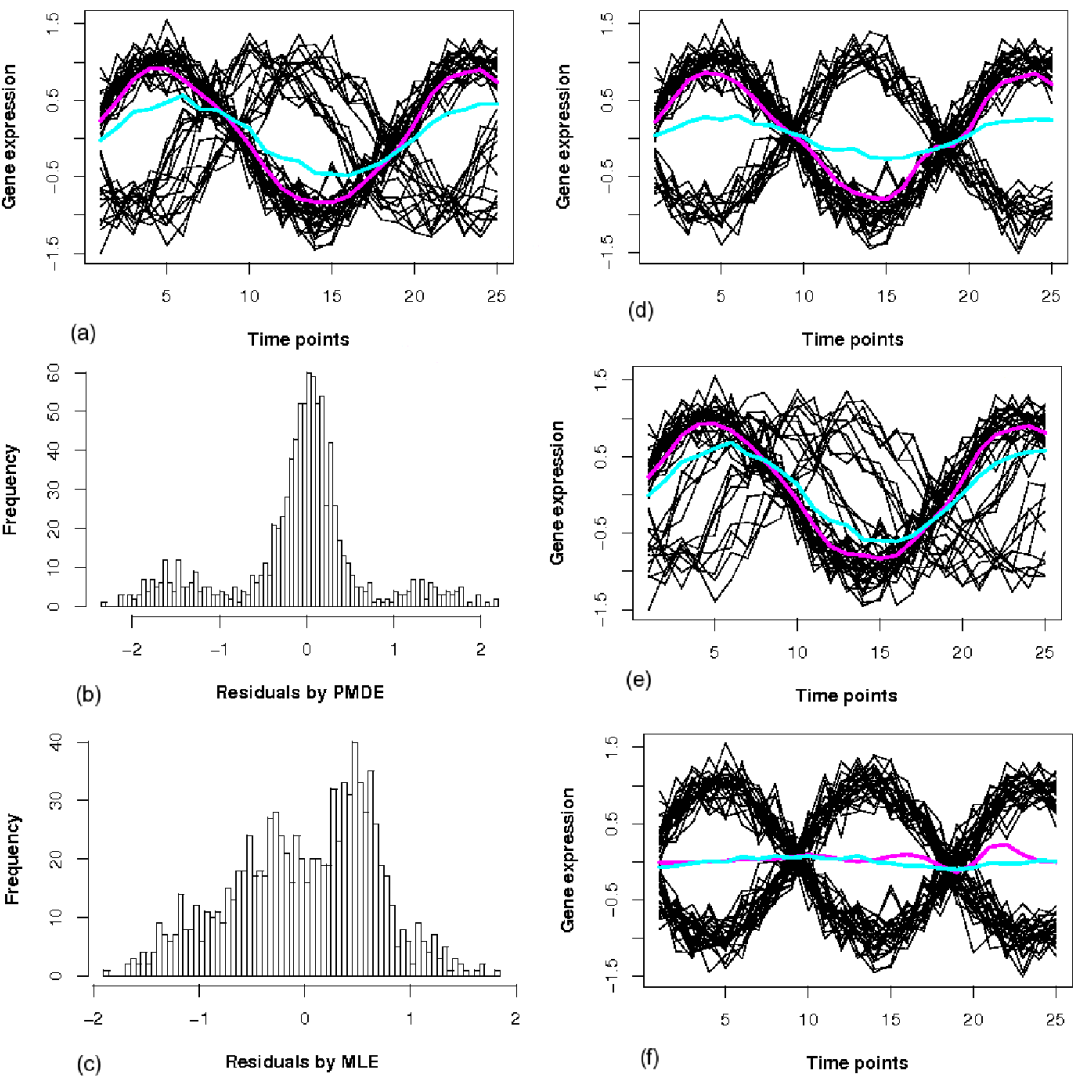
**Comparing PMDE and MLE by data fitting and their residual histograms**. (a) PMDE fit (pink line) and MLE fit (blue line) to simulated data generated from three sine waves; (b) Histogram of residuals by PMDE; (c) Histogram of residuals by MLE; (d) PMDE fit (pink line) and MLE (blue line) fit to simulated data generated from two sine waves; (e) PMDE fit (pink line) and MLE (blue line) fit to data with many outliers; (f) PMDE fit (pink line) and MLE (blue line) fit when two components are of same size.

We begin with simulating the situation when the number of components *K *(*K *= 3) is seriously underestimated as illustrated in Figure 1(a). Three components are generated from three sine waves simulating gene expression data of three clusters, each with 25 time points. The components comprise 60%, 20% and 20% of the data, respectively. The PMDE fit is highlighted by the pink line and the MLE fit is blue. PMDE locates the major component, while MLE is biased to all data. This is strong evidence that PMDE is superior to MLE in such a scenario. The fact that the PMDE can find the key component without compromising the others suggests a solution to the vexing problem when the number of components is unknown, which is often the situation in gene expression clustering. Histograms of residuals from both fits are plotted in Figure 1(b) and 1(c) to prove that PMDE fit the data in such a way that the residuals are close to normal.

More datasets shown in Figure 1(d)–(f) are used to compare the performances of PMDE and MLE in different scenarios. When there are two components of entirely opposite behaviors, we can see from Figure 1(d) that the MLE fit is almost flat, while PMDE fits the larger component (60% of the data). The situation where lots of outliers are present is simulated in Figure 1(e), where the major component has 60% of the data and the rest (40%) are generated from three different sine waves. PMDE demonstrates its robustness by capturing the major component, while MLE is seriously biased. However, in the case of two clusters of exactly equal size as shown in Figure 1(f), PMDE fails, as it is designed to capture only one component but now cannot decide which one to fit. This can be solved by using a multi-component model.

From these examples, it is observed that PMDE has the ability to handle the relevant fraction of data and distinguish it from outliers, while MLE blurs the distinction by accounting for all data. This is of great value for massive datasets, when the data structure is unclear and lots of outliers are present. The smoother fits of the proposed PMDE than that of MLE manifest the fact that the former is more robust against noise. All these suggest PMDE a promising tool for microarray data analysis. Interested readers are referred to Additional file 1, Section 1, for comparison of the two estimators on theoretical ground.

### Clustering Algorithm

When analyzing gene expression time-course data, special attention needs to be paid to the following issues:

• **Replicates**: It is desirable that the algorithm can naturally incorporate replicate information instead of simply using the mean of all replicates.

• **Number of clusters**: The choice of *K *is always a problem. The categorization of supervised and unsupervised schemes are usually determined by how *K *is defined. In our unsupervised algorithm, new cluster generation automatically terminates when no new cluster can be found in the data.

• **Scattered genes**: Recently, many have proposed allowing a noisy set of genes not being clustered [8,14]. In microarray experiments, it is generally expected that, because of the nature of data and the existence of high noise levels, many genes could show uncorrelated variations and are unrelated to the biological process under investigation. Forcing these genes into clusters will only introduce more false positives, resulting in distorted clusters and difficulty in interpretation.

Apart from the aforementioned issues, like other clustering methods, the proposed algorithm needs a stopping criteria. In this work, a statistical measure of partition quality, the Calinski and Harabasz (CH) index [27], is used as formulated in Eq.(14).

(14) $$CH(K)=\frac{BSS(K)/(K-1)}{WSS(K)/(n-K)}$$

where *BSS*() and *WSS*() are the between-cluster and within-cluster distances defined as

(15) $$BSS(K)=\frac{1}{2}\sum_{l=1}^{K}\sum_{x_{i}\notin C_{l},x_{j}\in C_{l}}d^{2}(x_{i},x_{j})$$

(16) $$WSS(K)=\frac{1}{2}\sum_{l=1}^{K}\sum_{x_{i},x_{j}\in C_{l}}d^{2}(x_{i},x_{j})$$

*C*_*l *_in Eq.(15) and (16) stands for the *l*th cluster. The idea behind the CH measure is to compute the pairwise sum of squared errors (distances) between clusters and compare that to the internal sum of squared errors for each cluster. In effect, it is a measure of between-cluster dissimilarity over within-cluster dissimilarity. The optimum clustering outcome should be the one that maximizes the CH index in Eq.(14). The CH index was originally meant for squared Euclidean distance. Since the residuals are a natural product of our spline regression model, we use the their absolute value as distance measurement in *BSS*(*K*) and *WSS*(*K*) but without the square form.

#### Partial regression clustering algorithm

Tight clustering, by definition, builds compact clusters upon an existing partition. The initial partition, if not available, can be obtained by some empirical knowledge or heuristic clustering methods such as k-means. Given an initial partition, the clustering procedure is formulated as in Algorithm 1.

In the initialization step of the algorithm, an existing partition of a dataset is provided as input. The tightness threshold, *υ*, which controls the tightness and the number of the refined clusters produced by the algorithm as output, is defined as the reciprocal of the weighted mean variance of the clusters of the initial partition. Therefore, the greater the threshold is (i.e., the smaller the variance is), the tighter the clusters become and the more clusters are formed. The weights are determined in proportional to the size of the clusters. In the main loop, after each new cluster is

**Algorithm 1 **Partial Regression Clustering

**Require: **Initialization

   **repeat**

      1. Fit partial regression model to each of the clusters;

      2. Identify potential outliers according to a tightness threshold *υ *and discard them from the clusters;

      3. For all outliers, fit partial regression model to form a new cluster;

      **repeat**

         4. For all genes re-evaluate distances to all existing spline regression models, assign them to the closest one;

         5. Fit partial regression models to all clusters;

         6. Calculate CH value based on current partitions;

      **until **the clustering quality measured by CH value fails to improve.

      7. Take the partition with highest CH value.

   **until **no partial regression model can be fitted to the outliers.

   8. Label all outliers as scattered genes.

generated, all data points are reassigned in the gene redistribution loop, so resultant clusters should be of reasonable size. The rationale supporting our design is based on the features of partial modelling and robustness of the MDE estimator, which we believe is able to find the relevant components in the data, while not being distracted by outliers. The residuals, as a natural byproduct of model fitting, can be used as the distance between data points and spline regression models.

In this framework, we use deterministic class assignment during the clustering process. Stochastic relaxation or weighted assignment is regarded as more moderate than deterministic assignment. However, it is also commonly recognised that stochastic relaxation, such as simulated annealing, does not guarantee convergence. In fact, the selection of starting temperature or the setting of annealing schedule are often heuristic. An initial temperature, set too high, leads to high computational cost while an initial temperature, set too low, yields similar result as deterministic relaxation but incurs higher computational cost than deterministic relaxation. After intensive testing with stochastic and deterministic relaxation on the datasets we used, we observed that deterministic assignment strikes a better balance between computational cost and clustering accuracy.

### Experiment on Simulated Dataset

Simulated datasets are necessary in evaluating the algorithm performance because the biological meanings of real datasets are very often not clear. Besides, simulated datasets provide more controllable conditions to test an algorithm. To obtain a meaningful result, the simulated data need to share statistical characteristic with biological data.

A simulated dataset is generated from a model *x*(*i, j*) = *α*_*i *_+ *β*_*i*_*ψ *(*i, j*) + *ε *(*i, j*), where *ψ *(*i, j*) = sin(*γ*_*i*_*j *+ *ω*_*i*_). *α*, *β*, *γ*, *ω *are cluster-specific parameters and are chosen according to the normal distribution with mean equal to 2 and standard deviation 1. All pattern details are listed [see Additional file 1, Section 2]. *ψ *models the cyclic behavior of gene expression patterns. 30 time points are taken from 6 of these models, so *i *∈ 1, 2, ..., 6, *j *∈ 1, 2, ..., 30. The cluster sizes are 50, 60, 70, 80, 90, 80. To model the noisy environment of microarray experiments, Gaussian noise *ε *is added to all data, together with 10 outliers generated by adding large variance Gaussian noise to three sine waves. Altogether, the simulated dataset is of size 440. Finally, we made some perturbations to induce more ambiguity, such as reducing the amplitude of parts of the patterns.

The clustering results are depicted in Supplementary Figure 1 of Additional File 1. The correct partition is achieved, with all ten outliers detected as shown in the seventh plot and the whole dataset plotted in the last one.

### Experiments on Yeast Cell Cycle (Y5) Dataset

A clustering method can be evaluated on theoretical grounds by internal or external validation, or both. For internal validation, a statistical measure is preferred. Our algorithm is first validated via the CH measure in a comparison with SplineCluster and MCLUST, two of the most popular clustering methods in the literature. On the other hand, a measure of agreement such as the adjusted Rand index (ARI) [28] between the resulting partition and the true partition, if known, is often used as an external validation criterion. Although a lot of evaluations for methods of the same kind are conducted in this way [8,26,29,30], we note that there is currently no ground truth, given our knowledge of the biological structures [31]. Recognizing this, we set out to evaluate the performance of our algorithm through systematically finding biologically relevant evidence [32-34]. The key to interpret a clustering outcome is to recognize the functional relationships among genes within a cluster as well as between clusters. We first provide a quantitative measure based on the graph structure of Gene Ontology, then pursue biological validation and inference through GO enrichment analysis in an empirically way.

#### Clustering Y5 dataset

A subset of 384 genes in Yeast *Saccharomyces Cerevisiae *Cell Cycle (Y5) dataset [26,35] measured at 17 time points was previously clustered [36] into five clusters based on the first peak time in the cell cycle: Early G1(G1E), late G1(G1L), S, G2 and M phase. The original partition, as shown in Supplementary Figure 2 of Additional File 1], indicates ambiguities between groups. Note that this dataset is chosen not only because it is well-studied in the gene expression clustering literature, but also because of its difficulty in terms of clustering. The original partition makes use of only partial information of gene expression which partly explains why many clustering algorithms have poor performance (ARI lower than 0.5 when it is used as external index [30,37]). The biological structure is still unclear, even in such heavily investigated organisms as Yeast *Saccharomyces Cerevisiae*. Moreover, the average cluster size (see the right most column of Table) is still far larger than desirable for efficient biological inference. It was recently suggested that clustering based on overall profiles is preferred to the original partition on a different subset from the same dataset [33]. We employ the proposed partial regression clustering algorithm to partition the Y5 dataset into tight clusters. By obtaining tighter clusters, we expect to obtain more informative and efficient biological inference. The tightness threshold *υ *is set to 8 as a result of estimation during the initialization and the number of knots for the spline basis is set experimentally to 13 to allow flexibility of the curve without overfitting.

**Figure 2:**
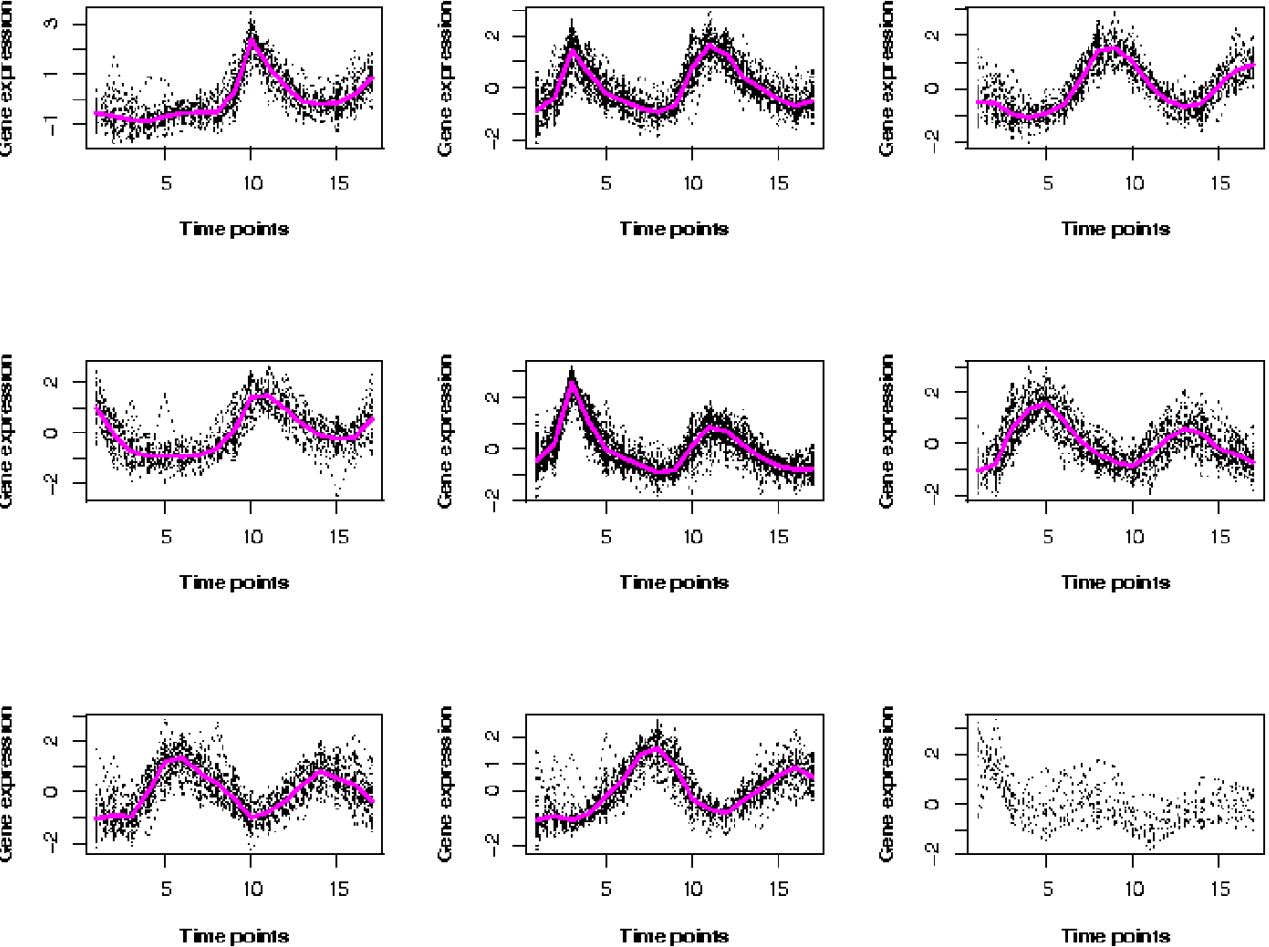
**The resulting clusters by the partial regression clustering algorithm for Y5 dataset**. The bottom right plot are the scattered genes.

The clustering outcome of our algorithm is plotted in Figure 2. Genes in the bottom right plot are the scattered genes. The eight clusters (C1–C8) with scattered genes (SG) are then cross-tabulated with the original partition in Table 1. The bottom row indicates the sizes of clusters of our partition and the right-most column shows those of the original partition. The two partitions agree on many genes but also differ in a interesting way. Our partition reveals neat and easily differentiable patterns. Also, we examined the clustering outcome given by our algorithm and by other algorithms.

**Table 1:** Cross tabulation of original partition and resulting partition for Y5 dataset.

	C1	C2	C3	C4	C5	C6	C7	C8	SG	Total
G1E	29	2	12	19	3	0	0	0	2	67
G1L	5	52	0	10	63	4	0	0	1	135
S	1	8	0	2	18	33	11	1	1	75
G2	0	0	0	0	0	7	30	10	5	52
M	1	0	23	0	0	0	1	29	1	55

Total	36	62	35	31	84	44	42	40	10	384

The left-most column contains the original partition and the top row has the resulting partition, C1–C8 are the eight clusters and SG are the set of scattered gene. Each number in the table except the right-most column and bottom row is the number of genes in both clusters corresponding to its row and column.

**Figure 3:**
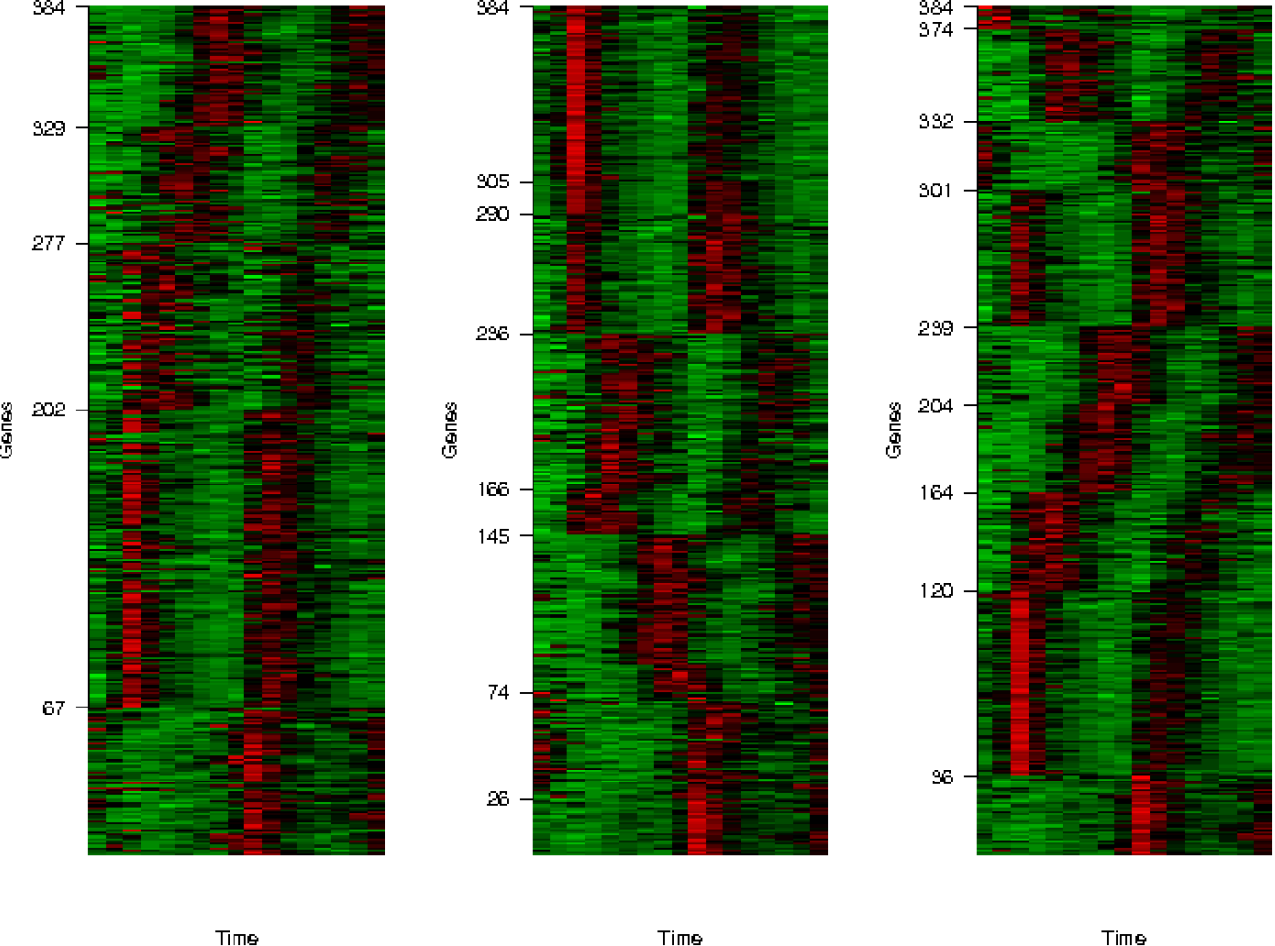
**Heatmaps for original partition (left), SplineCluster (middle) and the proposed algorithm (right)**. Brighter red color corresponds to higher expressions and brighter green color corresponds to lower expressions.

First of all, to see the effect of scattered gene detection, three algorithms are compared based on the full dataset (384 genes). By controlling a parameter in SplineCluster we obtained 8 clusters for comparison. The partitions of original, SpineCluster and partial regression analysis are illustrated in heatmaps plotted in Figure 3 for comparison, where an obvious improvement with respect to class distinction can be seen in the last heatmap. The tick marks on vertical axis in each heatmap indicate where the clusters are located, while in the last heatmap the last (top) cluster corresponds to the scattered genes. The second original cluster which is split into the sixth, seventh, and eighth clusters in the SplineCluster partition, and the second and fifth cluster in our partition. A closer look at the seventh and eighth cluster in the SplineCluster partition shows they differ only slightly in the peak values. However, in microarray data analysis, distinct expression patterns are more interesting than different peak values. This is one of the reasons we use a spline model in our algorithm to capture biologically relevant information. Consider the third cluster in the SplineCluster partition, which is split into the sixth and seventh clusters in our partition. The two clusters show two entirely different patterns, one shifted from the other. From these results, it is obvious that because of its ability in scattered gene detection, our algorithm reveals more distinguishable patterns in the data. The set of scattered genes is listed in Supplementary Table 1 of Additional File 2 with their annotations.

Then we use the 374 genes (excluding the 10 scattered genes), and again obtained 8 clusters for SplineCluster. As there is no biological knowledge input, comparison can first be conducted in a purely statistical manner, by the CH index. MCLUST [38] is a widely used mixture model-based clustering method. It is unsupervised, not only in determining the number of clusters, but also in selecting the type of model that best fit the data. The R implementation of MCLUST is used in our experiment. For the 374-gene dataset it decided on the EEE (Equal volume, shape and orientation) model and also found 8 components. Our algorithm achieves the highest CH value of 637.4, followed by 588.3 by MCLUST and 523.3 by SplineCluster.

#### Gene ontology enrichment analysis

To investigate how genes within a cluster are functionally related, and how clustering helps distinguish such functional groups, we apply Gene Ontology (GO) enrichment analysis to our clustering outcome. GO terms that are likely to be over-represented in each of the clusters are identified. These GO terms are of interest because they represent the most common functions that the genes in a cluster share. The probability that a given functional class is over-represented in the gene clusters can be estimated by using the hypergeometric distribution [39]. First, for each cluster, all unique GO terms that are associated with the genes in the cluster are identified. Then for each term two statistics are needed: the number of genes in the cluster that are annotated at each term and all known genes annotated at each term. With this information, the hypergeometric distribution can be applied to identify GO terms that are associated to more genes than by chance. The probability is indicated by the resultant *p*-values. Using the hypergeometric distribution, suppose there are *j *genes annotated to a function in a total of *G *genes in the genome, the *p*-value of observing *h *or more genes in a cluster of size *b *annotated to this function is given by

(17) $$p[O\geq h]=1-\sum_{i=0}^{h-1}\left(\begin{array}{c} b\\ i \end{array}\right)\left(\begin{array}{c} G-b\\ j-i \end{array}\right)/\left(\begin{array}{c} G\\ j \end{array}\right)$$

The lower the *p*-value is, the more unlikely the null hypothesis that the terms appear by chance is true. In this way, the over-represented terms are found for each cluster.

**Figure 4:**
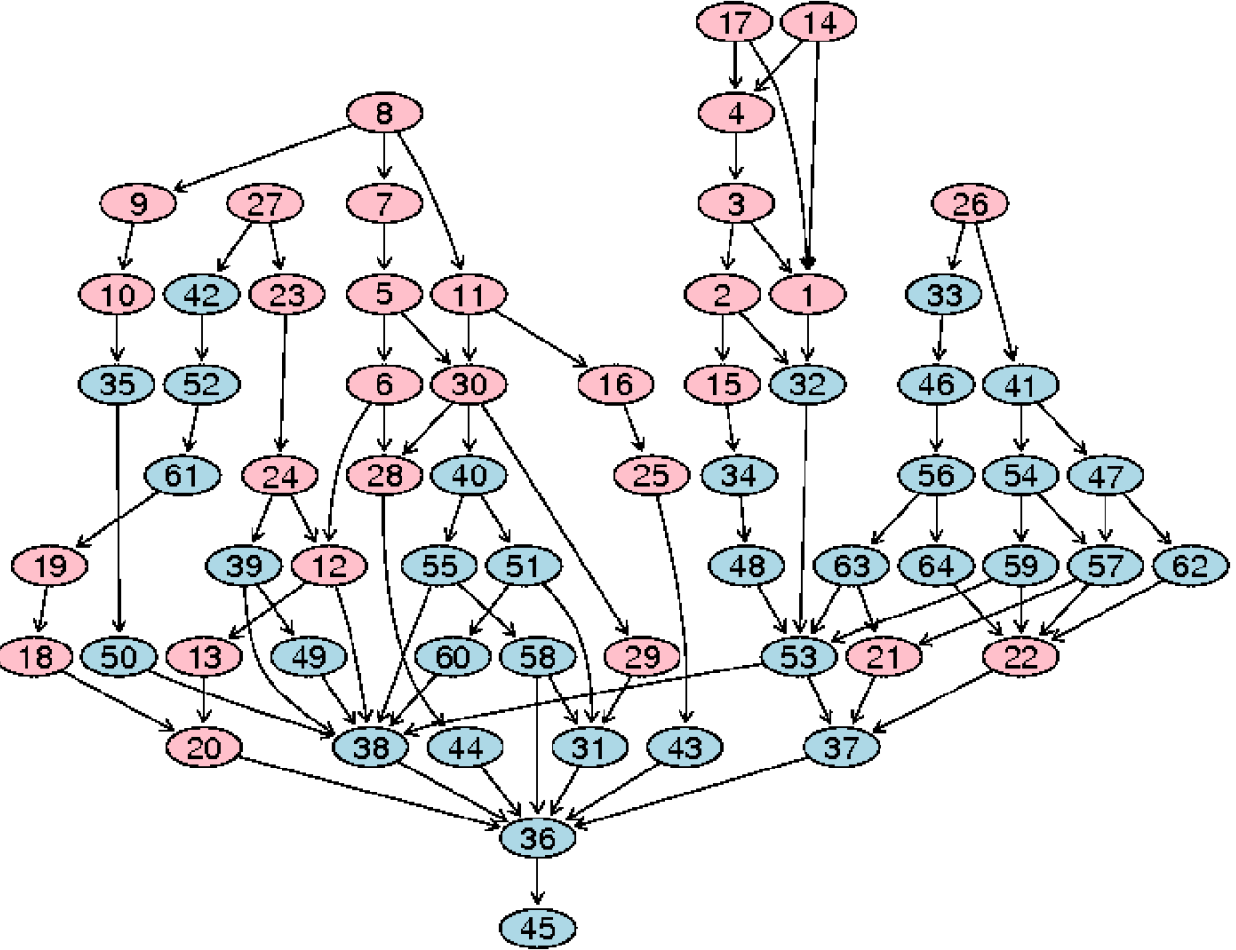
**An example of the GO tree graph**. Each number in a node can be mapped to a GO term in the corresponding GO term table (Supplementary Table 2 of Additional File 2). The over-represented terms are marked as pink, listed in the table together with their *p*-values and counted number of associated genes.

We propose within-cluster compactness (WCC) to measure the functional closeness for genes within one cluster based on the corresponding GO relationship graph. For each cluster *C*_*l*_, *l *∈ {1, 2, ..., *K*}, the most over-represented GO terms *T*_*l *_= {*t*_1_, *t*_2_, ..., *t*_*nl*_} are found, together with their corresponding p-values *P*_*l *_= {*p*_1_, *p*_2_, ..., *p*_*nl*_}. A GO relationship graph *G*_*l *_can be plotted using *T*_*l *_as input, linking to their parents until the root 'Biological Process' is reached. This measure aims to encourage deeper graphs with lower *p*-values while discouraging terms in different subgraphs with low *p*-values. For example, the GO graph in Figure 4 has two big subgraphs with their node details and p-values listed in Supplementary Table 2 of Additional File 2. The measure should be able to represent the large distance between nodes of different subgroups (e.g. node 1 and node 6) and their significance in terms of their p-values. Therefore, we define the GO distance between two terms as *D*_*ij *_= *d*(*t*_*i*_, *t*_*j*_) × (-*log*_10 _(*p*_*i*_)) × (-*log*_10 _(*p*_*j*_)), where *d*(*t*_*i*_, *t*_*j*_) is the shortest path between two terms in GO graph and *D*_*i·*_= *d*(*t*_*i*_, *root*) × (*log*_10 _(*p*_*i*_)^2^) is the distance between a term and the root. As two terms can share parents via multiple paths, the shortest distance between two terms in a GO graph is defined as the shortest path by which the two terms reach a shared parent, the lowest common ancestor (LCA). The sum of such distances for all paired GO terms can be used to indicate how closely the terms are related within a cluster. Thus, within-cluster compactness for a cluster *C*_*l *_is defined as

(18) $$WCC(C_{l})=\frac{\sum_{t_{i}\in T_{l}}D_{i}.}{\sum_{t_{i}\in T_{l}}\sum_{t_{j}\in T_{l},j\neq i}D_{ij}}$$

**Figure 5:**
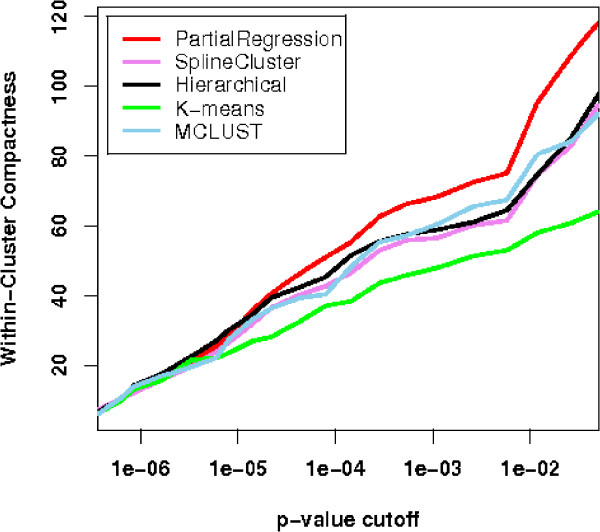
**Within-cluster compactness for five clustering algorithms for Y5 dataset**. Five clustering algorithms are assessed by their scores in terms of within-cluster compactness. The results are plotted against different *p*-value cut-offs. The higher the curve the better the performance of the algorithm is.

The sum of WCC for all clusters can then serve as a measure for a clustering outcome in terms of its compactness of cluster representation of biological functions. Five clustering algorithms: partial regression, SplineCluster, MCLUST, hierarchical clustering, and K-means are compared by pooling results, using different *p*-value cut-offs. Using the notion of false discovery rate (FDR) [40], adjusted *p*-values are used in accordance to confidence levels, for example 2% of FDR means accepting all tests with adjusted *p*-values < 0.02 as significant. The performances of different algorithms are relatively consistent (Figure 5), revealing a certain robustness of this measure. Our partial regression algorithm has the highest functional cluster closeness among the five methods, indicating superior performance. To explain what leads to such different yet consistent WCC scores and how the scores reconcile with biological knowledge, we analyse the functional categories that are statistically over-represented in the clusters. First, we compared the over-represented terms in the resulting clusters of the proposed algorithms (PMDE clusters) and SplineCluster (SC clusters). For simplicity, we based the following analysis in the Biological Process Ontology (Supplementary Table 3 and Supplementary Table 4 [see Additional File 2]). As indicated by the lowest P-values in each cluster, all PMDE clusters have a statistically significant set of cell cycle related terms (lowest *P *< 10^-5^), while for SC only six out of eight clusters have such significance. We observed that from the remaining two clusters of poorer quality (*P *= 6.35 × 10^-3 ^and 2.51 × 10^-4^), some genes involved in DNA replication (*SLD2*,*POL12*, *CDC45 *etc. [36]) were combined into PMDE cluster 5, resulting in a tight cluster that has a significantly functional over-representation of DNA strand elongation (*P *= 5.04 × 10^-9^) and other functions in DNA replication. Such a high quality cluster is essential for predicting unknown functions of genes such as *YHR151C *and *YNL058C *within the cluster. In addition, good agreement was found between known biological functions and gene clusters found by the proposed algorithm. Many clusters are significantly enriched with distinctive cell cycle relevant functions, indicating a good separation of functional clusters. For example, cluster 5 has an over-representation of DNA strand elongation (*P *< 10^-8^) and cluster 6 is enriched with microtubule nucleation and chromosome segregation (*P *< 10^-7^) which is crucial to chromosome division. Consistent with their biological functions, two clusters involving genes expressed in M and earlier phases reveal patterns of slightly different peak time: cluster 3 contains an over-representation of genes involved in DNA unwinding during replication (*P *< 10^-8^) and DNA geometric change (*P *< 10^-7^); and cluster 8 is enriched with cytokinesis that is known to occur after replication and segregation of cellular components. The two gene patterns are both biologically meaningful and statistically sound.

#### Predictive accuracy

We compared five clustering methods: our partial regression algorithm, SplineCluster, MCLUST [38], hierarchical clustering, K-means, in terms of their predictive accuracy established in [8]. Since the underlying biological ground truth is unknown, evaluation of clustering algorithms for gene data cannot be carried out by similarity measures such as ARI. Instead, predictive accuracy was proposed to test functional prediction accuracy from clustering. The rationale is that since clustering is aimed at functional prediction of novel genes, if a cluster has exceptionally high occurrences of a certain gene annotation *F *(*p*-value smaller than a certain threshold), all genes in this cluster can be predicted to be in the functional category *F*. The ratio of the verified predictions to all prediction made reflects the accuracy of a clustering algorithm. However, we have to bear in mind that this measure greatly depends on the annotation quality of the dataset under study.

Since our results involved a set of scattered genes, we propose as described below a slightly different criterion to the one in [8]. Suppose a functional category, *F*_*i*_, has *v*_*i *_genes in a dataset of size *n*. If there are in total *V *genes belonging to functional categories *F*_1_, *F*_2_, ..., *F*_*M*_, the remaining *n *- *V *genes are denoted as 'unannotated'. Such grouping and the resulting partition *C*_1_, *C*_2_, ..., *C*_*K *_of a clustering method can be cross-tabulated to form a table. Let *n*_*ij*_, (*i *= 1, 2, ..., *M *and *j *= 1, 2, ..., *K*) be the (*i*, *j*) entry of the table denoting the number of annotated genes, *p*_*ij *_be the corresponding *p*-value, and *n*_·*j *_be the size of cluster *C*_*j*_. Given a threshold *δ*, for a *K*-cluster solution, its predictive accuracy *A *is defined as

(19) *A*(*δ*) = *P*_*V *_(*δ*)/*P*_*C *_(*δ*)

where *P*_*V *_(*δ*) is the verified predictions and *P*_*C*_(*δ*) is the predictions calculated by

$$\begin{array}{c} P_{V}(\delta )=\sum_{j=1}^{K}\sum_{i\in \{x|p_{xj}<\delta \}}n_{ij}\\ P_{C}(\delta )=\sum_{j=1}^{K}\sum_{i\in \{x|p_{xj}<\delta \}}n._{j} \end{array}$$

**Figure 6:**
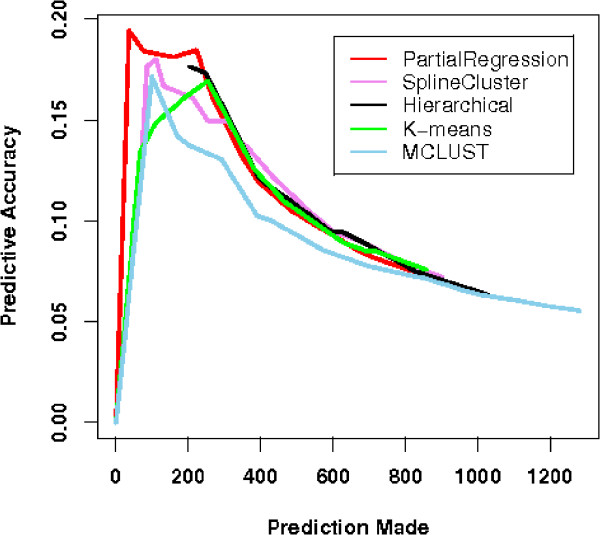
**Predictive accuracy plots for five clustering methods on Y5 dataset**. Five clustering methods are evaluated in terms of their functional group prediction accuracy. The five methods are partial regression(red), SplineCluster(violet), MCLUST(black), hierarchical clustering(green), K-means(blue). The higher the curve is the better the performance.

Supplementary Table 5 of Additional File 2 lists 68 genes in Y5 dataset that are verified to be cell cycle related to their corresponding cell cycle phase, together with their annotations. The 68 genes along with the remaining 316 genes denoted as 'unannotated' can then be cross-tabulated with our partition as in Supplementary Table 6 [see Additional file 2]. The bottom row of Supplementary Table 6 shows the size of clusters and the set of scattered genes. All scattered genes are excluded from this evaluation. By pooling results from various thresholds, we obtain a curve of 'prediction made' versus 'accuracy' for each method in comparison (*K *= 8). As shown in Figure 6, the curve for our partial regression method is above the others, indicating higher accuracy in functional group prediction.

#### Scattered genes

Another important aspect in our investigation is to study the set of scattered genes. Multiple experiments are conducted with various tightness thresholds, *υ*, in our partial regression method. In Supplementary Table 1 of Additional File 2 the set of scattered genes found in eight runs of our program with various thresholds and their annotations are presented. Their frequencies of appearance in these experiments are shown in the column Feq. (out of 8). We noticed that although these thresholds result in different numbers of clusters, the set of scattered genes hardly changes (Supplementary Table 1, column Feq.). Such consistency leads one to think about the underlying biological meaning. As has already been pointed out [2], scattered genes can be those individuals that are not relevant to the biological process under study. However, we stress here that they can also be of significant interest, as each of them might be a key component of the cell cycle that may affect other components and indeed may be a transcription factor themselves. Therefore, its expression pattern can be uncorrelated to others in the set under study. Alternatively, a scattered gene can represent a gene whose expression is controlled by more transcription factors than the other co-regulated genes within clusters. Moreover, because the set of genes under investigation is usually selected after performing gene ranking, there may be others in the complete list that would cluster with scattered genes. All these considerations drove us to further investigate this set of scattered genes.

**Figure 7:**
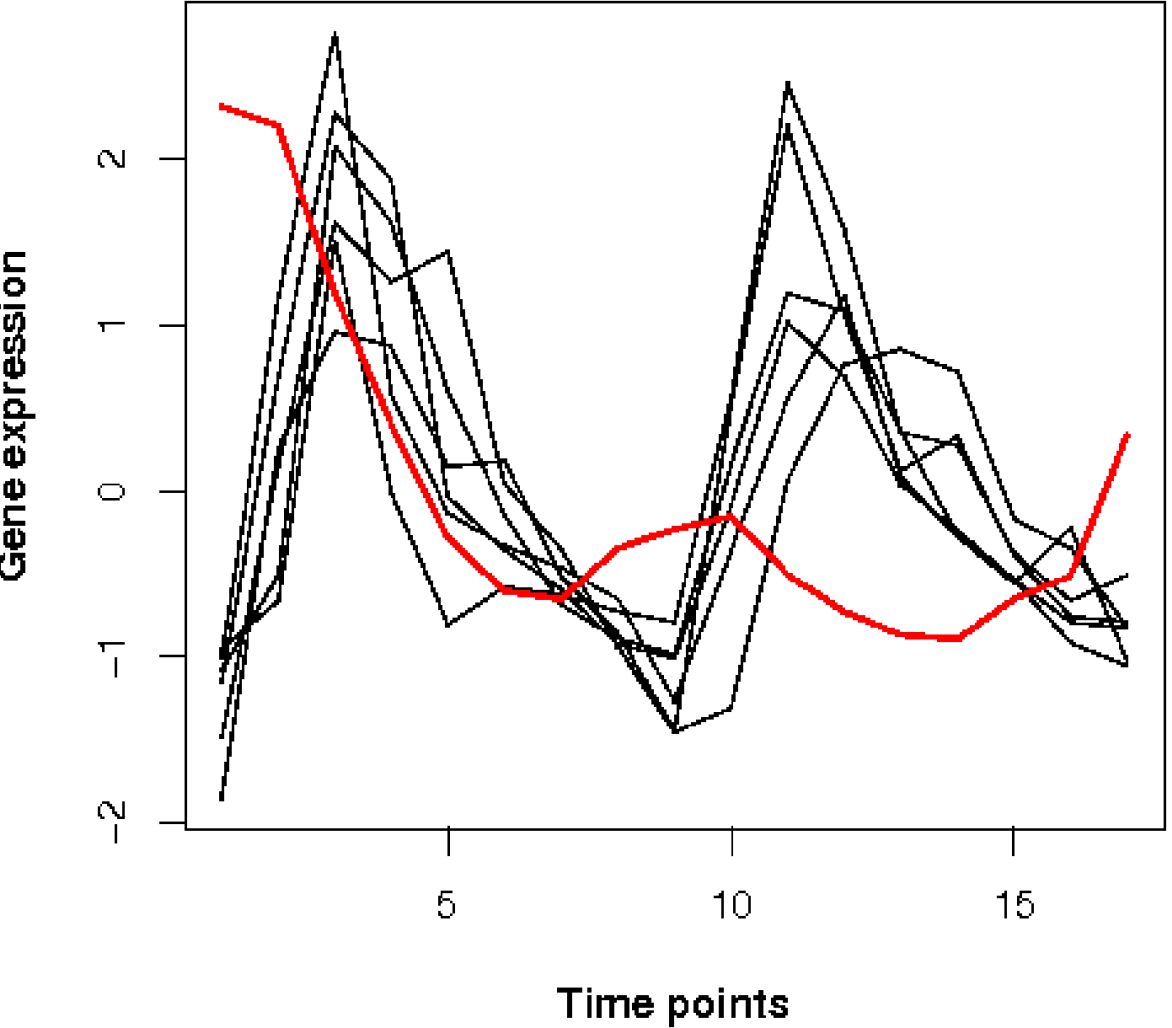
**The profiles of seven genes related to Late G1, SCB regulated cell cycle phase**. The red profile is the gene 'TIP1/YBR067C', one of the ten scattered genes. It displays a distinctive pattern from the other six genes annotated to be in the same functional group.

Among the scattered genes, five are either not well-understood or unknown for their functions. Only one of them, *TIP1/YBR067C*, is verified to be cell cycle related in phase Late G1, SCB regulated (Supplementary Table 5 of Additional File 2, second group). Indeed, according to Supplementary Table 5, one would conclude that all the seven genes in Late G1, SCB regulated phase to have the same behaviour. However, when their profiles are plotted as in Figure 7, we can see that *TIP1/YBR067C *is uncorrelated to the others, making it an interesting subject for further study.

#### Comparative evaluation on scattered gene detection

**Figure 8:**
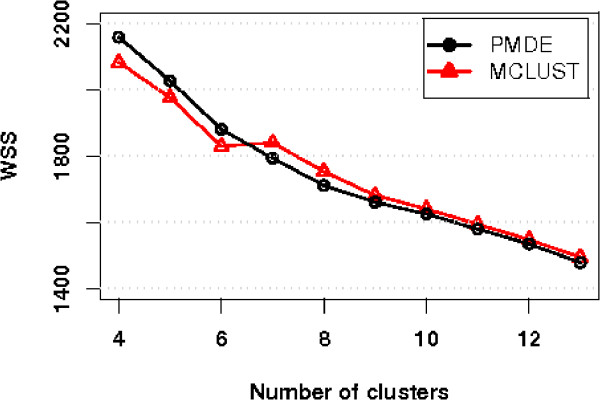
**Comparison of performance of PMDE and MCLUST in outlier detection**. A small index value of *WSS *indicates better performance in outlier filtering. PMDE performs better than MCLUST with large number of clusters.

To further assess the proposed PMDE's strength of scattered gene detection, the proposed algorithm is compared with a recent modification of the MCLUST, which allows an additional component of homogeneous Poisson process for scattered genes/noise [41]. The idea is for each method to filter out scattered genes and then, instead of analysing the scattered genes, compare the quality of the filtered datasets in terms of within-cluster sum of squares *WSS *as defined in Eq.(16). If an algorithm is stronger in outlier filtering, tighter clusters should be found in the filtered dataset, hence a smaller value of *WSS*. Since the number of scattered genes identified by the two methods may vary, when the sets of scattered genes filtered out by different methods are of different sizes, we randomly sample a subset of the same size as the smaller set from the lager one and return the leftovers to the filtered dataset so that the filtered datasets to be investigated/clustered are of the same size. Because the clustering quality may be affected by the returned genes, we repeat the process of the random sampling of scattered genes and the clustering of the filtered dataset 10 times, and take the average value of *WSS *to compare against the *WSS *of the clustering result by the other method. We obtain clustering results with the number of clusters *K *ranging from 4 to 13 for Y5 dataset from both the PMDE and the MCLUST. The results are plotted in Figure 8. We can see that the proposed PMDE performs better with large number of clusters, *K*, but not as good as the MCLUST with smaller *K*. However, this does not mean that the MCLUST outperforms the PMDE because the PMDE is designed to start with an initial set of clusters and iteratively split the current clusters if the splitting can lead to tighter clusters. Therefore, the clustering results by the PMDE with smaller values of *K *are not "final" but just "provisional"; when compared to the "final" results by the MCLUST, the performance of the PMDE appears to be inferior. However, when the results by the PMDE is more mature as *K *gets bigger, for example when *K *is greater than or equal to 7 as shown in Figure 8, the proposed PMDE consistently outperforms MCLUST.

### Experiments on Yeast Galactose dataset

Experiments are conducted on the Yeast Galactose dataset [42], which consists of gene expression measurements in galactose utilization in Saccharomyces cerevisiae. Gene expression was measured with 4 replicate assays across 20 experimental conditions (20 perturbations in the GAL pathway). A subset of measurements of 205 genes whose expression patterns reflect four functional categories in the GO listings was chosen and clustered previously [17,29]. Compared with Y5 dataset, Yeast Galactose dataset show more distinguishable patterns, which is easier for clustering and leads to more agreeable correlation to its functional interpretation.

**Figure 9:**
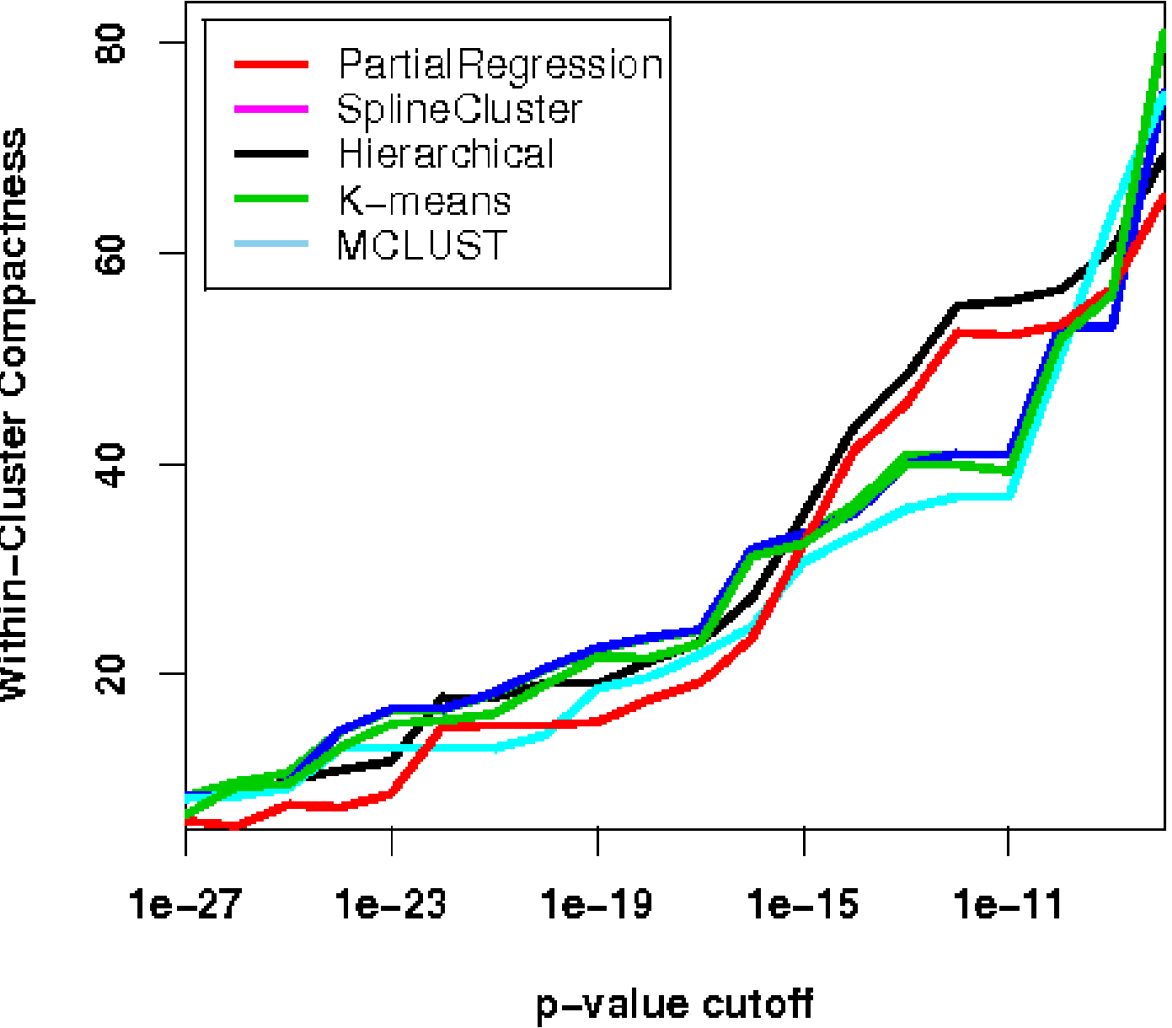
**Within-cluster compactness for five clustering algorithms for Yeast Galactose dataset**. For Yeast Galactose dataset, the plot of WCC scores for five clustering algorithms against different *p*-value cut-offs indicates best performance for the proposed algorithm.

For this dataset, our partial regression algorithm takes as input all 4 replicates of microarray data, yielding 4 clusters with 4 scattered genes when the tightness threshold is set to low value. The four clusters (C1–C4) with scattered genes (SG) are then cross-tabulated with the original partition in Table 2. We take 4 as cluster number, since it is also in accordance with prior knowledge, and obtain partitions from all five algorithms. Following, the results of WCC measure from five algorithms are plotted in Figure 9 across different *p*-value cut-offs. Consistent with previous findings [17,29], the WCC curves in Figure 9 show that most of the algorithms performed well on this dataset. The result from partial regression algorithm excels in both biological and statistical validation. After the scattered genes are excluded by partial regression, the average of WCC scores across different cut-offs are 27.5, 26.4, 26.4, 24.3, and 26.6, for partial regression, Spline Cluster, Hierarchical, K-means, and MCLUST, respectively. As a mean of statistical validation, CH measure is applied to the above five algorithms, giving values of 365.6, 331.1, 360.1, 255.3, and 364.5, respectively.

**Table 2:** Cross-tabulation of original partition (O1–O4) and resulting partition (C1–C4 and SG) for Yeast Galactose dataset.

Cluster	O1	O2	O3	O4	Total
C1	83	0	0	0	83
C2	0	12	0	0	12
C3	0	1	90	1	92
C4	0	1	0	13	14
SG	0	1	3	0	4

Total	83	15	93	14	205

The bottom row contains cluster sizes for the original partition and the right-most column contains cluster sizes for the resulting partition. Each number in the table except the right-most column and bottom row is the number of overlapping genes in both clusters corresponding to its row and column.

**Figure 10:**
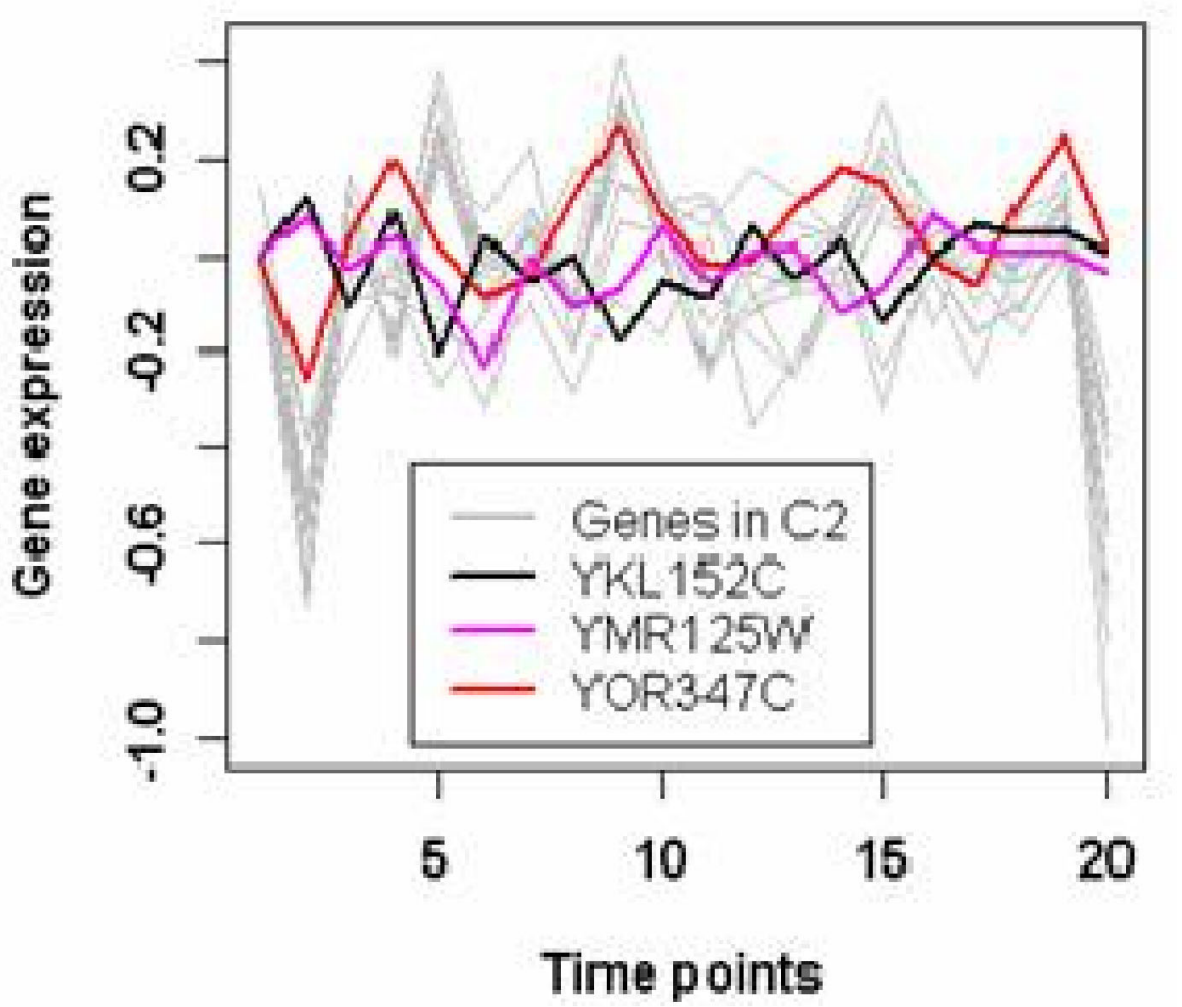
**Scattered genes in original cluster 2 of the Yeast Galactose dataset**. The expression profiles of some scattered genes detected by the proposed algorithm are plotted for the Yeast Galactose dataset. This plot shows the expression patterns of all 15 genes in original cluster 2, among them the 3 colored genes are the detected scattered genes;

Meanwhile, there are interesting findings from the investigation of scattered genes. For instance, one gene (*YMR125W*) belonging to the original cluster O2 is classified as a scattered gene. Of the other 14 genes in original cluster 2, 12 are clustered into C2, 1 in C3 (*YKL152C*) and 1 in C4 (*YOR347C*). The expression data of all of the 15 genes are plotted in Figure 10, revealing very different expression patterns of the 12 genes and the 3 genes differentiated by our algorithm. Both *YKL152C *and *YMR125W *are up-regulated at the beginning with down regulations for all others. The resulting cluster C2 by partial regression is verified by GO, since the 12 genes share similar annotations among the 15 genes in the original cluster O2, for example they are all annotated to Glycolysis (GO:0006096) observed from the Supplementary Table 7 of Additional File 2.

**Figure 11:**
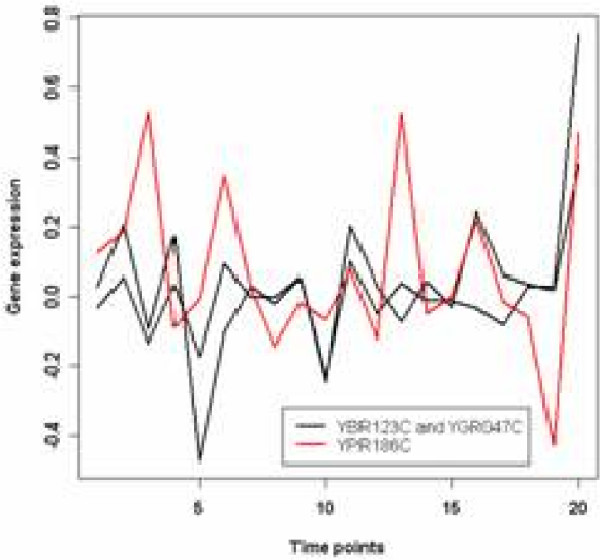
**Scattered genes in original cluster 3 of the Yeast Galactose dataset**. The expression profiles of the 3 scattered genes in original cluster 3. They share GO annotations but have various expression patterns.

As an important transcription factor, *YPR186C *is an essential protein that binds the 5S rRNA gene through the zinc finger domain and directs assembly of a multi-protein initiation complex for RNA polymerase III. Belonging to the original cluster O3, *YPR186C *is classified as a scattered gene. We plot its expression levels together with two other genes that are also annotated to GO:0006384 (transcription initiation from RNA polymerase III promoter), and found dramatic differences among their patterns in Figure 11. Since this term is quite specific and it should largely reflect a gene's function, mechanisms behind such diverse behaviours are still unclear and are worth further investigations. In summary, our algorithm receives highest WCC score. The validity of its partitions are proved through GO analysis. We expect that its ability of scattered gene prediction will be well sought after.

## Conclusion

The aim of clustering gene profiles is to find possible functional relationships among tens of thousands of genes on a microarray. We propose that while the models for data fitting should be sensitive enough for discriminating individuals/genes, the estimators should be robust enough against noise and possible outliers. Therefore we focused on the differences between estimators by providing experimental comparisons. The robustness of the minimum distance estimator makes it stand out in our study. An immediate advantage is that when it is applied to gene expression clustering, it is capable of locating the key components in an unsupervised manner. As a result, a set of scattered genes that has low correlations is naturally obtained. Besides the GO enrichment analysis for the clusters from two real datasets, inference of the sets of scattered genes was also highlighted in this paper.

The partial mixture model (PMM) was known to solve problems for low dimensional data. In fact, one problem with classical PMM is that it cannot fit data of more than 7 data points [13]. This is the first time PMM is extended to use on high dimensional data, since current microarray experiments are having more time points and more replicates. Our contributions include introducing MDE and the idea of partial modelling to gene expression research, giving comparisons with the most common estimator in the literature – maximum likelihood, and proposing a novel partial regression clustering algorithm. Our spline regression model captures the inherent time dependencies among data. The error term is of particular importance as it can pick up the noise. The fact that PMDE estimates parameters so the residuals are as close to normal distribution as possible makes it a powerful tool for modelling the error term. The tightness of resulting clusters can be controlled by a threshold which in a sense decides the number of clusters. The effectiveness of the algorithm also depends on the model normality. When model normality holds approximately, clusters can be found. Often gene expression data are transformed during pre-processing so that normality holds approximately.

Although many interactions between genes are known, our knowledge of biological networks is far from complete. No conclusion can be drawn by merely comparing clustering inference with known measure from the biological literature. In this case, we aim to validate the algorithm and explain the clustering outcome with the help of various biological resources. As a highlight of this paper, Gene Ontology clustering validation was applied to the clustering outcomes of Yeast cell cycle dataset and Yeast Galactose dataset. From current knowledge, it is proved that these clusters can help separate groups of genes with similar functions, while new information can be learned from exploring the GO terms. First we proposed a novel measure based on graph theory and annotation knowledge as functional compactness indication for clusters. Further, predictive accuracy was utilized to compare the annotation prediction power across several common methods. Both measures confirmed that our proposed method has the best performance. Also, gene annotations reveal new knowledge that can be derived from scattered genes. A concern about GO analysis and annotation is that lots of genes and their functions are still unknown or poorly understood. It is our hope that through clustering, new understanding can be introduced to genome research.

In summary, the proposed system benefits from the robustness of MDE to detect scattered genes, the idea of partial modelling for tight clusters, the spline regression model for capturing the expression curves at either uniformly or unevenly distributed time points, and the use of the design matrix for incorporating replicate information. The proposed algorithm can be applied over an existing clustering to get tighter clusters. Although PMDE demonstrates its effectiveness through comparisons with maximum likelihood method, it also has its limits such as relative inefficiency. The aim of this paper is not to prove which one is better, but rather to provide analytical examples, discussions and insights.

## Authors' contributions

YY conceived of the study, proposed the formulae, carried out the implementation and prepared the manuscript. C-TL revised the formulae and advised on the preparation of the manuscript. RW advised on the preparation of the manuscript. All authors have approved the final manuscript.

## Supplementary Material

Additional File 1 **Theoretical comparison between MDE and MLE**. Theoretical comparison between minimum divergence estimator and maximum likelihood estimator. **Simulated dataset patterns**. Patterns for generating simulated dataset. Supplementary Figure 1. The resulting clusters by the partial regression clustering algorithm for the simulated dataset. Supplementary Figure 2. The original partition of the Yeast Y5 dataset, bottom right plot is the whole dataset. Click here for file

Additional File 2 Supplementary Table 1. The set of scattered genes for Yeast Y5 dataset. Supplementary Table 2. Table for the GO relationship graph. Supplementary Table 3. Over-represented GO terms by Partial Regression Algorithm. Supplementary Table 4. Over-represented GO terms by SplineCluster. Supplementary Table 5. Verified cell cycle related (68) genes in Y5 dataset. Supplementary Table 6. Cross-tabulation of clustering outcome (C1–C8 and SG) with verified gene functional categories for Yeast Y5 dataset. Supplementary Table 7. Over-represented terms in each original cluster for Yeast Galactose dataset. Click here for file
