# Supplementary material for: Partial mixture model for tight clustering of gene expression time-course
Source: BMC Bioinformatics. 2008 Jun 18;9:287. doi: 10.1186/1471-2105-9-287 (PMC2492882; doi:10.1186/1471-2105-9-287)
Supplement: Additional File 1 — Theoretical comparison between MDE and MLE. Theoretical comparison between minimum divergence estimator and maximum likelihood estimator. Simulated dataset patterns. Patterns for generating simulated dataset. Supplementary Figure 1. The resulting clusters by the partial regression clustering algorithm for the simulated dataset. Supplementary Figure 2. The original partition of the Yeast Y5 dataset, bottom right plot is the whole dataset. [file 1471-2105-9-287-S1.pdf]

Supplementary File

March 12, 2008

1 Theoretical comparison between MDE and MLE

Both minimum distance estimator and maximum likelihood estimator belong to the Minimum distance (MD) family. Given the vector of parameters of interest $\theta_0 \in \Theta$ where Θ is the set of possible parameter values, MD estimator can be generalized as minimizing a criterion function:

$$F(\theta) = \hat{g}(\theta) D_w(\theta) \tag{1}$$

where $\hat{g}(\theta)$ is a function of the data y_t that will verify $\hat{g}(\theta_0) \to 0$, and $D_w(\theta)$ being a weighted distance matrix. Depending on the choices of $\hat{g}(\theta)$, different estimators can be generated.

In particular, a minimum divergence estimator, which incorporates minimum distance and maximum likelihood, is proposed [1] as an alternative to non-parametric density estimation. Density-based minimum divergence methods include those estimate parameters through minimizing some pre-defined divergence between the assumed model density and the true model density underlying the data, e.g. maximum likelihood method and minimum chi-squared method. The criterion is given by

$$\theta = \arg\min_{\theta} [\int f(x|\theta)^{1+\alpha} dx - \frac{1+\alpha}{n\alpha} \sum_{i=1}^{n} f(x_i|\theta)^{\alpha}] \tag{2}$$

with a metaparameter $\alpha > 0$. MDE corresponds to $\alpha = 1$ while MLE corresponds to $\alpha \to 0$.

An example of the two estimation criteria, for normal density $X \sim N(\mu, \sigma^2)$:

$$\hat{\mu}_{MLE} = \arg\max_{\mu} \sum_{i=1}^{n} \log \phi(x_i|\mu, \sigma^2) \tag{3}$$

$$\hat{\mu}_{MDE} = \arg\min_{\mu} (\frac{1}{2\sigma\sqrt{\pi}} - \frac{2}{n} \sum_{i=1}^{n} \phi(x_i|\mu, \sigma^2)) \tag{4}$$

While the aim of MDE is to maximize the sum of the densities, MLE tries to maximize the product of the densities.

2 Simulated dataset patterns

Simulated dataset is generated from the following patterns, with parameters generated from normal distributions:

$$
\begin{aligned}
x1(i,j) &= 0.1 + sin(1/3j) + \varepsilon(i,j) \\
x2(i,j) &= -0.1 + sin(1/3j - 1) + \varepsilon(i,j) \\
x3(i,j) &= 1.2sin(2/5j - 2) + \varepsilon(i,j) \\
x4(i,j) &= 1.5sin(1/3j - 3.5) + \varepsilon(i,j) \\
x5(i,j) &= 0.5sin(2/5j - 2.2) + \varepsilon(i,j) \\
x6(i,j) &= 0.6sin(1/3j - 3.8) + \varepsilon(i,j)
\end{aligned} \tag{5}
$$

3 Additional Figures

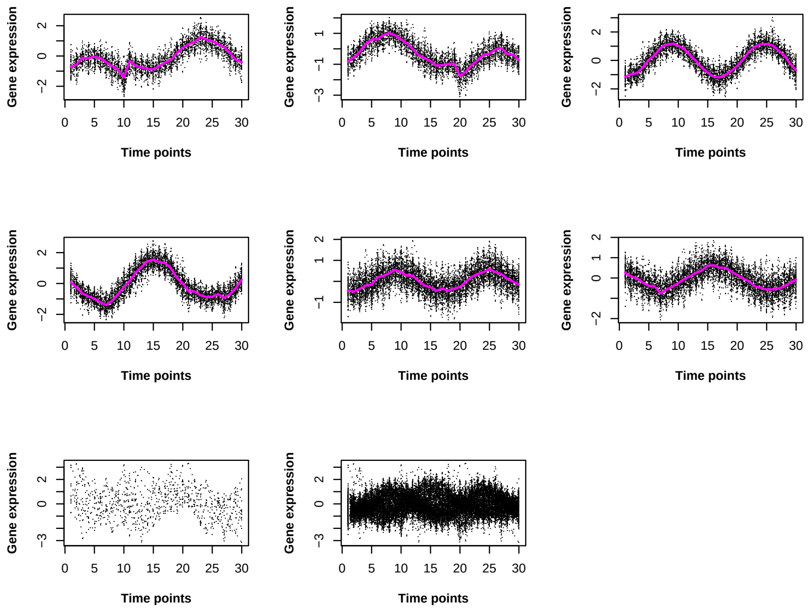

Figure 1: The resulting clusters by the partial regression clustering algorithm for the simulated dataset. The right plot in the third row is the whole dataset and the left plot are the outliers.

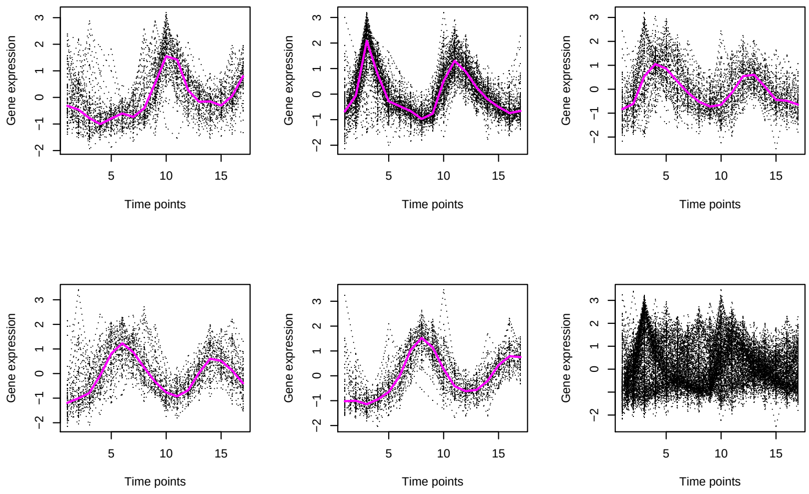

Figure 2: The original partition, bottom right plot is the whole dataset

References

[1] A. Basu, I.R. Harris, N.L. Hjort, and M.C. Jones. Robust and efficient estimation by minimising a density power divergence. *Biometrika*, 85:549–559, 1998.
