# Supplementary material for: Partial mixture model for tight clustering of gene expression time-course
Source: BMC Bioinformatics. 2008 Jun 18;9:287. doi: 10.1186/1471-2105-9-287 (PMC2492882; doi:10.1186/1471-2105-9-287)
Supplement: Additional File 2 — Supplementary Table 1. The set of scattered genes for Yeast Y5 dataset. Supplementary Table 2. Table for the GO relationship graph. Supplementary Table 3. Over-represented GO terms by Partial Regression Algorithm. Supplementary Table 4. Over-represented GO terms by SplineCluster. Supplementary Table 5. Verified cell cycle related (68) genes in Y5 dataset. Supplementary Table 6. Cross-tabulation of clustering outcome (C1–C8 and SG) with verified gene functional categories for Yeast Y5 dataset. Supplementary Table 7. Over-represented terms in each original cluster for Yeast Galactose dataset. [file 1471-2105-9-287-S2.doc]

**Supplementary Table 1. The set of scattered genes for Yeast Y5 dataset**

| Gene | Freq. | Function | Remark | Link to SGD database |
| --- | --- | --- | --- | --- |
| BRE2/ YLR015W | 7/8 | Subunit of the COMPASS (Set1C) complex, which methylates histone H3 on lysine 4 and is required in transcriptional silencing near telomeres |  | [SGD:S000004005](http://db.yeastgenome.org/cgi-bin/locus.pl?sgdid=S000004005) |
| MOD5/ YOR274W | 7/8 | Delta 2-isopentenyl pyrophosphate:tRNA isopentenyl transferase, required for biosynthesis of the modified base isopentenyladenosine in mitochondrial and cytoplasmic tRNAs |  | [SGD:S000005800](http://db.yeastgenome.org/cgi-bin/locus.pl?sgdid=S000005800) |
| PPR1/ YLR014C | 7/8 | Zinc finger transcription factor containing a Zn(2)-Cys(6) binuclear cluster domain, positively regulates transcription of genes involved in uracil biosynthesis |  | [SGD:S000004004](http://db.yeastgenome.org/cgi-bin/locus.pl?sgdid=S000004004) |
| RNP1/ YLL046C,  YNL016W | 8/8 | Ribonucleoprotein that contains two RNA recognition motifs (RRM) |  | [SGD:S000003969](http://db.yeastgenome.org/cgi-bin/locus.pl?sgdid=S000003969) |
| TIP1  /YBR067C | 8/8 | Major cell wall mannoprotein with possible lipase activity | Verified to be cell cycle related | [SGD:S000000271](http://db.yeastgenome.org/cgi-bin/locus.pl?sgdid=S000000271) |
| UIP4/ YPL186C | 8/8 | Protein of unknown function that interacts with Ulp1p, a Ubl (ubiquitin-like protein)-specific protease for Smt3p protein conjugates |  | [SGD:S000006107](http://db.yeastgenome.org/cgi-bin/locus.pl?sgdid=S000006107) |
| YBR184W | 8/8 | Putative protein of unknown function |  | [SGD:S000000388](http://db.yeastgenome.org/cgi-bin/locus.pl?sgdid=S000000388) |
| YDL124W | 8/8 | NADPH-dependent alpha-keto amide reductase |  | [SGD:S000002282](http://db.yeastgenome.org/cgi-bin/locus.pl?sgdid=S000002282) |
| YDR366C | 5/8 | Hypothetical protein |  | [SGD:S000002774](http://db.yeastgenome.org/cgi-bin/locus.pl?sgdid=S000002774) |
| YLL047W | 8/8 |  | Not annotated | SGD: S000003970 |

This table contains the details of 10 scattered genes, the frequencies that they are found across eight experiments of various thresholds, their annotations and links to SGD database.

Supplementary Table 2. Table for the GO relationship graph

| Node | GO | Genes | GO.Term | G.count | T.count | P-value |
| --- | --- | --- | --- | --- | --- | --- |
| 1 | GO:0006118 | CYT1; COX7; MCR1; QCR9; COX8 | electron transport | 5 | 33 | 0.000153 |
| 2 | GO:0006119 | CYT1; COX7; QCR9; COX8; INH1 | oxidative phosphorylation | 5 | 46 | 0.000266 |
| 3 | GO:0042773 | CYT1; COX7; QCR9; COX8 | ATP synthesis coupled electron transport | 4 | 25 | 0.000266 |
| 4 | GO:0042775 | CYT1; COX7; QCR9; COX8 | ATP synthesis coupled electron transport (sensu Eukaryota) | 4 | 25 | 0.000266 |
| 5 | GO:0031137 | MF(ALPHA)1; FAR1; MF(ALPHA)2; GPA1 | regulation of conjugation with cellular fusion | 4 | 26 | 0.000266 |
| 6 | GO:0046999 | MF(ALPHA)1; FAR1; MF(ALPHA)2; GPA1 | regulation of conjugation | 4 | 26 | 0.000266 |
| 7 | GO:0032005 | MF(ALPHA)1; FAR1; MF(ALPHA)2; GPA1 | signal transduction during conjugation with cellular fusion | 4 | 26 | 0.000266 |
| 8 | GO:0000750 | MF(ALPHA)1; FAR1; MF(ALPHA)2; GPA1 | pheromone-dependent signal transduction during conjugation with cellular fusion | 4 | 26 | 0.000266 |
| 9 | GO:0007186 | MF(ALPHA)1; FAR1; MF(ALPHA)2; GPA1 | G-protein coupled receptor protein signaling pathway | 4 | 32 | 0.000555 |
| 10 | GO:0007166 | MF(ALPHA)1; FAR1; MF(ALPHA)2; GPA1 | cell surface receptor linked signal transduction | 4 | 48 | 0.00255 |
| 11 | GO:0000749 | MF(ALPHA)1; FAR1; MF(ALPHA)2; GPA1 | response to pheromone during conjugation with cellular fusion | 4 | 56 | 0.00424 |

**Supplementary Table 3**. Over-represented GO terms by Partial Regression Algorithm

| Cluster |  | Ontology | P-values | Int counts | GO Counts |
| --- | --- | --- | --- | --- | --- |
| 1 | GO:0006118 | electron transport | 1.06E-06 | 5 | 33 |
| 1 | GO:0006119 | oxidative phosphorylation | 5.82E-06 | 5 | 46 |
| 1 | GO:0042775 | ATP synthesis coupled electron transport (sensu Eukaryota) | 1.13E-05 | 4 | 25 |
| 2 | GO:0006974 | response to DNA damage stimulus | 1.09E-06 | 12 | 226 |
| 2 | GO:0045005 | maintenance of fidelity during DNA-dependent DNA replication | 2.56E-06 | 5 | 24 |
| 2 | GO:0000135 | septin checkpoint | 3.37E-06 | 3 | 4 |
| 3 | GO:0006268 | DNA unwinding during replication | 3.31E-09 | 5 | 12 |
| 3 | GO:0032392 | DNA geometric change | 3.49E-08 | 5 | 18 |
| 3 | GO:0006270 | DNA replication initiation | 5.54E-07 | 5 | 30 |
| 4 | GO:0005975 | carbohydrate metabolic process | 7.61E-06 | 8 | 203 |
| 4 | GO:0006101 | citrate metabolic process | 0.000164 | 2 | 4 |
| 4 | GO:0006091 | generation of precursor metabolites and energy | 0.000185 | 7 | 235 |
| 5 | GO:0022616 | DNA strand elongation | 5.04E-09 | 8 | 30 |
| 5 | GO:0051276 | chromosome organization and biogenesis | 1.73E-08 | 26 | 556 |
| 5 | GO:0009719 | response to endogenous stimulus | 1.79E-08 | 17 | 235 |
| 6 | GO:0007020 | microtubule nucleation | 1.05E-08 | 6 | 23 |
| 6 | GO:0007017 | microtubule-based process | 2.92E-08 | 9 | 99 |
| 6 | GO:0007059 | chromosome segregation | 1.09E-07 | 9 | 115 |
| 7 | GO:0000070 | mitotic sister chromatid segregation | 3.84E-05 | 5 | 57 |
| 7 | GO:0007001 | chromosome organization and biogenesis (sensu Eukaryota) | 4.69E-05 | 13 | 551 |
| 7 | GO:0016481 | negative regulation of transcription | 5.08E-05 | 7 | 146 |
| 8 | GO:0000910 | cytokinesis | 2.14E-06 | 7 | 101 |
| 8 | GO:0000278 | mitotic cell cycle | 1.22E-05 | 9 | 244 |
| 8 | GO:0000916 | cytokinesis, contractile ring contraction | 0.000222 | 2 | 4 |

**Supplementary Table 4**. Over-represented GO terms by SplineCluster

| Cluster | GO Terms | Ontology | P-values | Int counts | GO Counts |
| --- | --- | --- | --- | --- | --- |
| 1 | GO:0006268 | DNA unwinding during replication | 7.38E-05 | 3 | 12 |
| 1 | GO:0006267 | pre-replicative complex formation | 9.54E-05 | 3 | 13 |
| 1 | GO:0050790 | regulation of catalytic activity | 0.000178 | 4 | 40 |
| 2 | GO:0006260 | DNA replication | 9.51E-08 | 10 | 112 |
| 2 | GO:0006310 | DNA recombination | 9.44E-07 | 9 | 110 |
| 2 | GO:0006974 | response to DNA damage stimulus | 9.14E-06 | 11 | 226 |
| 3 | GO:0022402 | cell cycle process | 1.63E-06 | 16 | 399 |
| 3 | GO:0000278 | mitotic cell cycle | 3.14E-05 | 11 | 244 |
| 3 | GO:0000074 | regulation of progression through cell cycle | 3.55E-05 | 9 | 162 |
| 4 | GO:0022616 | DNA strand elongation | 1.59E-10 | 9 | 30 |
| 4 | GO:0006273 | lagging strand elongation | 5.73E-09 | 7 | 20 |
| 4 | GO:0006261 | DNA-dependent DNA replication | 1.35E-07 | 9 | 91 |
| 5 | GO:0007165 | signal transduction | 0.006354 | 4 | 193 |
| 5 | GO:0007154 | cell communication | 0.010349 | 4 | 222 |
| 5 | GO:0030541 | plasmid partitioning | 0.011825 | 1 | 3 |
| 6 | GO:0009262 | deoxyribonucleotide metabolic process | 0.000251 | 2 | 8 |
| 6 | GO:0006259 | DNA metabolic process | 0.000476 | 7 | 503 |
| 6 | GO:0006334 | nucleosome assembly | 0.000587 | 2 | 12 |
| 7 | GO:0007017 | microtubule-based process | 9.30E-06 | 5 | 99 |
| 7 | GO:0007020 | microtubule nucleation | 4.25E-05 | 3 | 23 |
| 7 | GO:0009225 | nucleotide-sugar metabolic process | 9.01E-05 | 2 | 5 |
| 8 | GO:0007120 | axial bud site selection | 1.14E-06 | 5 | 21 |
| 8 | GO:0000819 | sister chromatid segregation | 1.66E-05 | 6 | 59 |
| 8 | GO:0000910 | cytokinesis | 3.97E-05 | 7 | 101 |

**Supplementary Table 5. Verified cell cycle related (68) genes in Y5 dataset**

| Cell Cycle | Genes Systematic Names |
| --- | --- |
| M/G1 Boundary | "YKL185W" "YLR274W" "YBR202W" "YJL194W" "YAL040C" "YLR286C" "YDL127W" "YDL179W" "YGR044C" "YLR079W" "YER111C" "YBR083W" |
| Late G1, SCB regulated | "YMR199W" "YPL256C" "YDL227C" "YER001W" "YNL289W" "YJL187C" "YBR067C" |
| Late G1, MCB regulated | "YJL115W" "YDL197C" "YOR074C" "YLR103C" "YDL164C" "YPR120C" "YGR109C"  "YPR175W" "YBR278W" "YDR309C" "YDL003W" "YOL090W" "YDR097C" "YKL101W" "YDR113C" "YNL082W" "YNL102W" "YBL035C" "YNL262W" "YBR088C" "YKL045W" "YKL113C" "YAR007C" "YNL312W" "YJL173C" "YER070W" "YDR356W" "YKL042W" "YPL153C" "YJL092W" "YML021C" |
| S-phase | "YBL003C" "YBL002W" |
| S/G2-phase | "YMR198W" "YPR141C" |
| G2/M-phase | "YLR131C" "YOR058C" "YGL116W" "YMR001C" "YGR108W" "YPR119W" "YGR092W" "YJL157C" "YAR018C" "YIL106W" "YBR054W" "YDR033W" "YHR152W" "YDR146C" |

**Supplementary Table 6**. Cross-tabulation of clustering outcome (C1-C8 and SG) with verified gene functional categories for Yeast Y5 dataset

| Cell Cycle | C1 | C2 | C3 | C4 | C5 | C6 | C7 | C8 | SG | Total |
| --- | --- | --- | --- | --- | --- | --- | --- | --- | --- | --- |
| M/G1 Boundary | 7 | 2 | 1 | 2 | 0 | 0 | 0 | 0 | 0 | 12 |
| Late G1, SCB regulated | 0 | 2 | 0 | 0 | 3 | 1 | 0 | 0 | 1 | 7 |
| Late G1, MCB regulated | 0 | 13 | 0 | 0 | 15 | 3 | 0 | 0 | 0 | 31 |
| S-phase | 0 | 1 | 0 | 0 | 0 | 1 | 0 | 0 | 0 | 2 |
| S/G2-phase | 0 | 0 | 0 | 0 | 0 | 1 | 1 | 0 | 0 | 2 |
| G2/M-phase | 2 | 0 | 3 | 1 | 0 | 0 | 0 | 8 | 0 | 14 |
| Not verified | 27 | 45 | 32 | 28 | 66 | 37 | 41 | 33 | 9 | 316 |
| Total | 36 | 62 | 35 | 31 | 84 | 44 | 42 | 40 | 10 | 384 |

This table contains cross tabulation of functional categories with a group of unnannotated gene and resulting clusters with a set of scattered genes.

**Supplementary Table 7. Over-represented terms in each original cluster for Yeast Galactose dataset**

| Term | Cluster | Ontology | pvalues | intcounts | goCounts | IC |
| --- | --- | --- | --- | --- | --- | --- |
| GO:0006412 | 1 | translation | 4.37E-95 | 83 | 372 | 0.437493 |
| GO:0044249 | 1 | cellular biosynthetic process | 1.46E-64 | 80 | 841 | 0.382007 |
| GO:0044260 | 1 | cellular macromolecule metabolic process | 4.96E-52 | 80 | 1192 | 0.323332 |
| GO:0019538 | 1 | protein metabolic process | 5.69E-52 | 80 | 1194 | 0.3211 |
| GO:0008152 | 1 | metabolic process | 4.85E-24 | 83 | 3064 | 0.241258 |
| GO:0006096 | 2 | glycolysis | 9.53E-29 | 12 | 22 | 0.60745 |
| GO:0019320 | 2 | hexose catabolic process | 1.22E-25 | 12 | 35 | 0.587928 |
| GO:0046164 | 2 | alcohol catabolic process | 2.24E-24 | 12 | 43 | 0.58356 |
| GO:0044275 | 2 | cellular carbohydrate catabolic process | 1.02E-22 | 12 | 57 | 0.564239 |
| GO:0006094 | 2 | gluconeogenesis | 3.06E-16 | 8 | 26 | 0.678861 |
| GO:0043170 | 3 | macromolecule metabolic process | 1.53E-35 | 92 | 2355 | 0.269566 |
| GO:0044238 | 3 | primary metabolic process | 3.99E-31 | 93 | 2763 | 0.250476 |
| GO:0044237 | 3 | cellular metabolic process | 6.52E-28 | 93 | 2988 | 0.247995 |
| GO:0000398 | 3 | nuclear mRNA splicing, via spliceosome | 9.31E-28 | 27 | 95 | 0.557452 |
| GO:0000375 | 3 | RNA splicing, via transesterification reactions | 1.07E-26 | 27 | 103 | 0.556128 |
| GO:0008643 | 4 | carbohydrate transport | 2.45E-26 | 12 | 35 | 0.595434 |
| GO:0008645 | 4 | hexose transport | 4.39E-25 | 11 | 26 | 0.663755 |
| GO:0051234 | 4 | establishment of localization | 7.64E-10 | 13 | 961 | 0.319579 |
| GO:0015766 | 4 | disaccharide transport | 0.002408395 | 1 | 1 | 1 |
| GO:0015771 | 4 | trehalose transport | 0.002408395 | 1 | 1 | 1 |
